# Recombinant Human IgG1-Hexamer Reduces Pathogenic Autoantibodies in the K/BxN Mouse Model of Arthritis Independent of FcRn

**DOI:** 10.3390/ijms27031277

**Published:** 2026-01-27

**Authors:** Bonnie J. B. Lewis, Ruqayyah J. Almizraq, Selena Cen, Beth Binnington, Kayluz Frias Boligan, Rolf Spirig, Fabian Käsermann, Shannon E. Dunn, Donald R. Branch

**Affiliations:** 1 Centre for Innovation, Canadian Blood Services, Keenan Research Centre, 30 Bond Street, Toronto, ON M5B 1W8, Canada; bonnie.lewis2@griffithuni.edu.au (B.J.B.L.); ralmizraq@bluerocktx.com (R.J.A.); selenacen1989@gmail.com (S.C.); beth.binnington@blood.ca (B.B.); kayluz@gmail.com (K.F.B.); 2 Department of Laboratory Medicine and Pathobiology, University of Toronto, 30 Bond Street, Toronto, ON M5B 1W8, Canada; 3 CSL Behring Biologics Research Center, 3014 Bern, Switzerland; rolfspirig@cslbehring.com (R.S.); fabian.kaesermann@cslbehring.com (F.K.); 4 Swiss Institute for Translational and Entrepreneurial Medicine, sitem-insel, 3010 Bern, Switzerland; 5 Sunnybrook Research Institute, 2075 Bayview Street, North York, ON M4M 2M5, Canada; shannon.dunn@utoronto.ca; 6 Department of Medicine, University of Toronto, 30 Bond Street, Toronto, ON M5B 1W8, Canada

**Keywords:** autoimmunity, B cells, antibodies, arthritis, K/BxN mouse, Fcγ receptor, neonatal Fc receptor, human recombinant Fc hexamer

## Abstract

Arthritis in K/BxN mice is provoked by pathogenic autoantibodies to glucose-6-phosphate isomerase (G6PI), which is a ubiquitously expressed enzyme that is present in cells, in the circulation and on articular cartilage. When G6PI autoantibodies (auto-Abs) deposit on the articular cartilage of K/BxN mice, arthritis ensues due to the activation of various components of the innate immune system. Recent studies have investigated the in vivo efficacy of recombinant fragment-crystallizable (Fc) protein-based therapeutics. Many recombinant Fc proteins evaluated provide protection against inflammation in mouse models of arthritis, such as the K/BxN serum-transfer model. More recently, rFc-µTP-L309C, a recombinant human IgG1-Fc with an additional point mutation at position L309C fused to the human IgM tailpiece to form a hexamer, has been shown to ameliorate the arthritis in K/BxN mice. Additional studies have shown that rFc-µTP-L309C has multiple effects that work together to ameliorate the arthritis, including inhibition of neutrophil migration into the joint, inhibition of IL-1β production, downregulation of Th1 and Th17 cells, and increases in T regulatory cells and synovial fluid IL-10. In this work, rFc-µTP-L309C was shown to effectively prevent arthritis in the K/BxN serum-transfer model, significantly downregulate inflammatory cytokines/chemokines, and ameliorate the arthritis in the endogenous K/BxN model. This amelioration of the arthritis was associated with a significant decrease in autoantibody levels, which was independent of the neonatal Fc receptor (FcRn). rFc-µTP-L309C was shown to specifically inhibit G6PI autoantibody secretion from B-cells with a concomitant increase in TGFβ and decrease in B-cell activating factor (BAFF). These new findings suggest that rFc-µTP-L309C may provide a therapeutic benefit for other antibody-mediated autoimmune diseases through its effects on B-cells.

## 1. Introduction

There is a need for therapies for antibody-mediated autoimmune diseases that affect antibody production itself. Antibody-mediated autoimmune diseases can lead to the formation of immune complexes (ICs) that are not cleared from the circulation and become highly pathogenic by engaging Fcγ receptors (FcγRs) to recruit and activate immune cells, activating the complement and inducing a local inflammatory response [1,2,3]. Therefore, it has been suggested that a blockade of these innate immune pathways, including the effector functions mediated by Fcγ receptors, can be a promising treatment for antibody-mediated autoimmune diseases [3,4]. The endogenous K/BxN mouse model of rheumatoid arthritis (RA) is a useful tool to understand how autoantibodies drive the progression of arthritis by interacting with downstream components of the innate immune system [5].

K/BxN mice are generated by breeding KRN mice expressing a TCR transgene for glucose 6-phosphate isomerase (G6PI) peptide in the context of IAg7 MHC class II expressed by NOD/Lt mice [5,6,7]. The F1 progeny demonstrate a severe inflammatory response driven by auto-anti-G6PI antibodies against the ubiquitously expressed self-antigen, G6PI, which is present in cells, in the circulation and on the articular cartilage [7]. When the G6PI autoantibodies deposit on the articular cartilage of K/BxN mice, arthritis ensues due to the formation of ICs that can activate various components of the innate immune system [8]. Although the role of autoantibodies in the pathogenesis of RA remains controversial, the form of arthritis that K/BxN mice exhibit is similar to that experienced by humans [7]. As in humans, the disease is chronic, progressive, and symmetrical, and it exhibits all the classical histological features such as leukocyte invasion, synovitis, pannus formation, cartilage, and bone destruction [9].

Recent studies have investigated the in vivo efficacy of recombinant Fc protein-based therapeutics. Various approaches for controlled multimerization of Fc to form polyvalent molecules have been explored [10]. Fusion of the human IgG2 hinge region to human IgG1 Fc or mouse IgG2a Fc led to expression of multimerized Fcs that bound FcγR with high avidity [11]. These molecules demonstrated therapeutic efficacy in animal models of arthritis with doses as low as ∼50 mg/kg body weight. Using an alternative strategy, Ortiz et al. [12] studied Fc multimers of increasing valency and identified molecules that bound FcγRs with high avidity without triggering activating signals. A trivalent molecule termed Fc3Y showed protection in mouse models of arthritis [12]. Finally, a recombinant human hexameric Fc molecule showed increased binding to FcγRs and effectively interfered with FcγR function [13]. Additionally, a similar molecule was also very effective at treating animal models of arthritis using passive antibody transfer [14].

A hexameric recombinant Fc multimer called rFc-µTP-L309C was produced by fusing the 18 AA IgM tailpiece to the *C*-terminus of a variant human IgG1 Fc with a point mutation at position 309 [4]. This point mutation facilitates the stabilized hexamerization of this molecule through the formation of disulfide e bonds [4]. We showed that rFc-µTP-L309C has high binding avidity for FcγRs and could suppress arthritis in the collagen-induced arthritis (CIA), collagen-antibody-induced arthritis (CAIA), and in the K/BxN mouse models [4,14]. We have also shown that treatment with Fc-µTP-L309C in the endogenous K/BxN mouse model of chronic RA results in inhibition of neutrophil migration into the joint and IL-1β production from the neutrophils [15]. Pretreatment with rFc-µTP-L309C of the K/BxN endogenous arthritis completely prevents the development of arthritis and cartilage damage [15]. Recently, we have looked at the effect of rFc-µTP-L309C on the T-cell compartment in the amelioration of arthritis in the K/BxN model and showed that treatment with rFc-µTP-L309C inhibits Th2 and Th17 T-cells and increases Tregs and IL-10 [16]. Thus, rFc-µTP-L309C has multiple effects on K/BxN arthritis involving neutrophils and T-cells. Here, we investigated what effects, if any, rFc-µTP-L309C has on B-cells and antibody production in this mouse model of RA. We show that pathogenic anti-GPI autoantibody levels in K/BxN mice are reduced upon repeated injection of rFc-µTP-L309C. This reduction in the atherogenic autoantibody does not involve antagonism of the neonatal Fc receptor (FcRn). It results from a direct effect on individual B-cell antibody secretion. The observed effect was presumably due to a local increase in TGF-β and a decrease in BAFF in the joints of K/BxN mice.

## 2. Results

### 2.1. Fc-µTP-L309C Lowers Anti-G6PI Ab Levels and Ameliorates Arthritis Independent of FcRn

**Figure 1:**
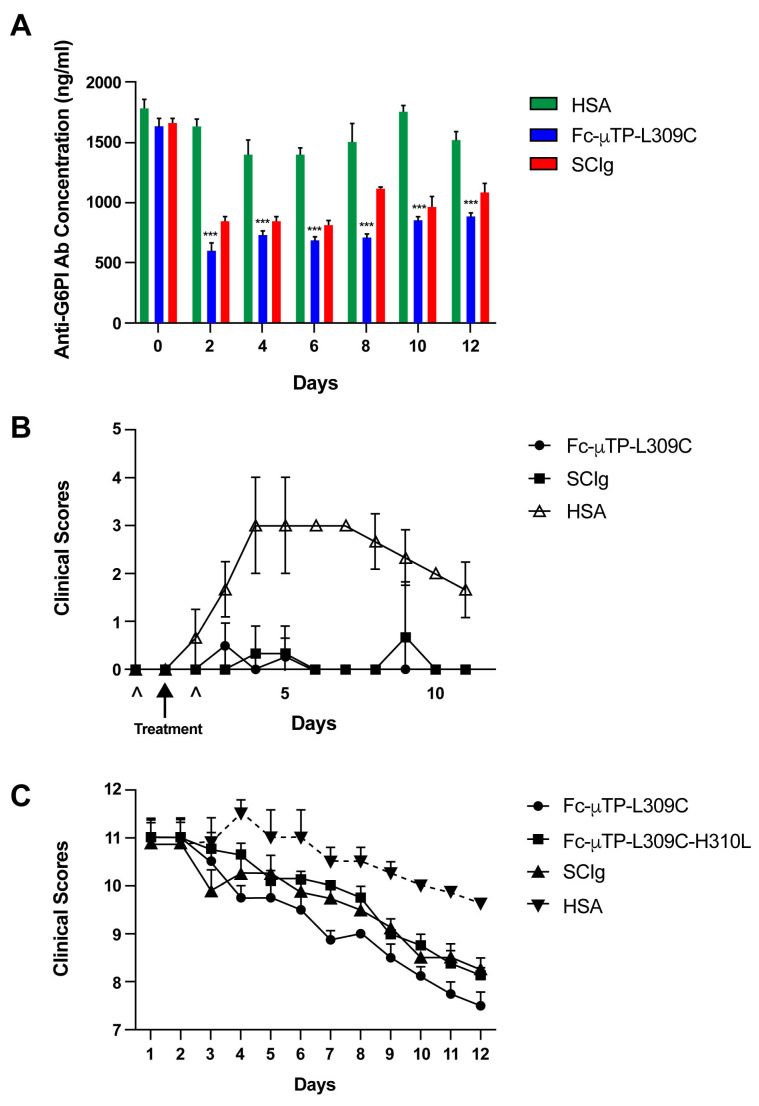
Fc-µTP-L309C ameliorates arthritis and reduces anti-G6PI Abs in K/BxN mice that are independent of FcRn. (**A**) Fc-µTP-L309C inhibits arthrogenic antibody production. Shown are the concentrations of anti-G6PI antibodies in the peripheral blood of K/BxN mice that were treated with 6 s.c. injections of either 200 mg/kg of Fc-µTP-L309C or 2000 mg/kg of SCIg, and HSA was used as a protein control. Injections were performed on days 1, 3, 5, 7, 9, and 11 and blood collection was performed on days 0, 2, 4, 6, 8, 10, and 12. Shown are the average anti-G6PI concentrations measured in ng/mL and the error bars indicate the range of concentrations (mean ± SD, *n* = 8). *** *p* ˂ 0.05, one-way ANOVA with Dunnett test, compared with HSA. (**B**) Fc-µTP-L309C prevents serum-transfer arthritis that is independent of FcRn. Clinical scores for serum-transfer-induced arthritis are shown for FcRn KO mice given i.p. injections of 200 µL of arthritic serum on days 0 and 2, indicated by ^, that were treated with 200 mg/kg of Fc-µTP-L309C or with 2000 mg/kg of SCIg on day 2, indicated by arrow, in comparison to mice treated with HASA. Shown are the average clinical scores; error bars indicate range of clinical scores (mean ± SD, *n* = 4). (**C**) Fc-µTP-L309C ameliorates endogenous K/BxN arthritis that is independent of FcRn. K/BxN mice given 6 s.c. injections of 200 mg/kg of Fc-µTP-L309C, 200 mg/kg of Fc-µTP-L309C-H310, or with 2000 mg/kg of SCIg on days 1, 3, 5, 7, 9, and 11. Shown are the average clinical scores; error bars indicate range of clinical scores (mean ± SD, *n* = 4). *** *p* ˂ 0.001 compared with HSA, Kruskal–Wallis with Dunn test.

K/BxN mice that were at high clinical scores of 9–12 were given six subcutaneous (s.c.) injections on days 1, 3, 5, 7, 9, and 11 of either 200 mg/kg of Fc-µTP-L309C or 2000 mg/kg of subcutaneous immunoglobulin (SCIg) (positive control treatment), and human serum albumin (HSA) was used as a protein control. The anti-G6PI Ab levels in the peripheral blood of these mice were measured on days 0, 2, 4, 6, 8, 10, and 12 by ELISA. The anti-G6PI Ab levels in the peripheral blood of K/BxN mice were lowered significantly over time in mice that received six s.c. injections of 200 mg/kg of Fc-µTP-L309C and in mice that received six s.c. injections of 2000 mg/kg of SCIg in comparison to mice that received HSA as a control (Figure 1A).

FcRn KO mice were injected intraparitoneally (i.p.) with 200 µL of arthritic sera on days 0 and 2. Mice were given treatment on day 2 with either a s.c. injection of 200 mg/kg of Fc-µTP-L309C or a s.c. injection of 2000 mg/kg of SCIg; HSA was used as a protein control. FcRn KO mice that were treated with HSA developed arthritis, whereas those receiving one s.c. injection of 200 mg/kg of Fc-µTP-L309C or 2000 mg/kg of SCIg did not (Figure 1B)

K/BxN mice with high clinical scores of 9–12 were treated by either s.c. injections of 200 mg/kg Fc-µTP-L309C, s.c. injections of 200 mg/kg Fc-µTP-L309C-H310L (H310L abrogates binding to FcRn), or s.c. injections of 2000 mg/kg of SCIg as indicated in the figure legends on days 1, 3, 5, 7, 9, and 11. HSA was used as a protein control. Fc-µTP-L309C-H310 ameliorated arthritis to a similar degree as Fc-µTP-L309C, as indicated by a decrease in clinical scores relative to those in the HSA group (Figure 1C).

### 2.2. Fc-µTP-L309C Reduces Autoantibody Production and Deposition in K/BxN Mice

**Figure 2:**
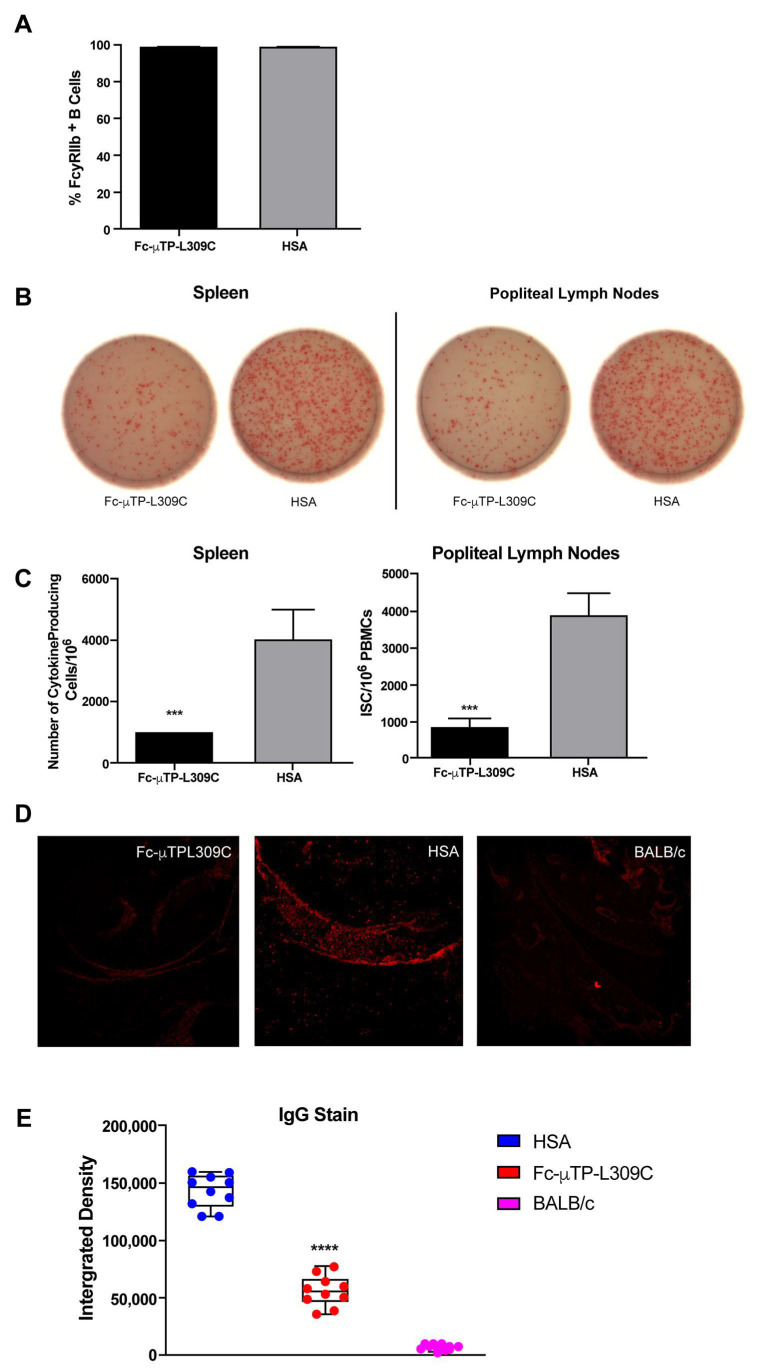
Fc-µTP-L309C does not bind to FcγRIIb on B-cells but does reduce anti-G6PI antibody production by B-cells and IgG deposits in joints of K/BxN mice. Shown are the percentages (**A**) of B-cells that stained positive for FcγRIIb from the spleens of K/BxN mice that were treated ex vivo with 100 µg/mL of Fc-µTP-L309C or with HSA. Shown are the average percentages; error bars indicate range of percentages (mean ± SD, *n* = 8). A representative well image (**B**) from one mouse for each organ with each treatment is shown. The immunoglobulin-secreting cells (ISCs)/106 PBMCs (**C**) are shown for the spleen and popliteal lymph nodes of K/BxN mice that were given 6 s.c. injections of 200 mg/kg of Fc-µTP-L309C, and HSA was used as a protein control (mean ± SD, *n* = 8). *** *p* ˂ 0.001 compared with HSA, Mann–Whitney test. Representative images (400x magnification) (**D**) of ankle sections from K/BxN mice that were given 6 s.c. injections of 200 mg/kg of Fc-µTP-L309C. Mice treated with HSA and BALB/c mice were used as controls. Ankle sections were stained with anti-IgG in red. (**E**) The average IgG deposition expressed as an integrated density (the product of area and mean gray value) is shown; error bars indicate the range of integrated densities of IgG depositions (mean ± SD, *n* = 10). **** *p* ˂ 0.0001 compared with HSA, Kruskal–Wallis with Dunn test.

Spleens were dissected from K/BxN mice and treated ex vivo with 100 µg/mL of Fc-µTP-L309C to analyze FcγRIIb staining on B-cells. A total of 99% of B-cells from spleens treated with Fc-µTP-L309C stained positive for FcγRIIb (Figure 2A), and no difference was observed from the group treated with HSA (protein control). Overall, no modulation of expression of FcγRIIb on B-cells in splenocytes was observed with Fc-uTP-L309C.

K/BxN mice that were at high clinical scores of 9–12 were given six s.c. injections on days 1, 3, 5, 7, 9, and 11 of 200 mg/kg of Fc-µTP-L309C, and HSA was used as a protein control. The spleens and popliteal lymph nodes from these mice were isolated on day 12 and anti-G6PI Ab production from B-cells in these organs was measured using ELISpot. There was significantly less anti-G6PI production in the spleens and popliteal lymph nodes in K/BxN mice that received six s.c. injections of 200 mg/kg of Fc-µTP-L309C in comparison to mice that received HSA (Figure 2B,C).

K/BxN mice that were at high clinical scores of 9–12, were given six s.c. injections on days 1, 3, 5, 7, 9, and 11 of 200 mg/kg of Fc-µTP-L309C, and HSA-treated mice. BALB/c mice were used as controls. The ankle joints of these mice were dissected on day 12 and stained for IgG deposition on the synovial lining using immunofluorescence microscopy. This deposition was quantified using ImageJ, Version 1.51w, Biotechne, Toronto, ON, Canada. There was significant IgG deposition on the synovial lining of the ankle joint of K/BxN mice that were treated with HSA (Figure 2D,E). There was significantly less IgG deposition on the synovial lining of the ankle joints of K/BxN mice that were treated with six s.c. injections of 200 mg/kg of Fc-µTP-L309C (Figure 2D,E). There was no IgG deposition on the synovial lining of the ankle joints of BALB/c mice, a non-diseased control (Figure 2D,E).

### 2.3. Multiplex Cytokine Analysis of Cytokine/Chemokines Associated with Inflammation in Peripheral Blood and Synovial Fluid

**Figure 3:**
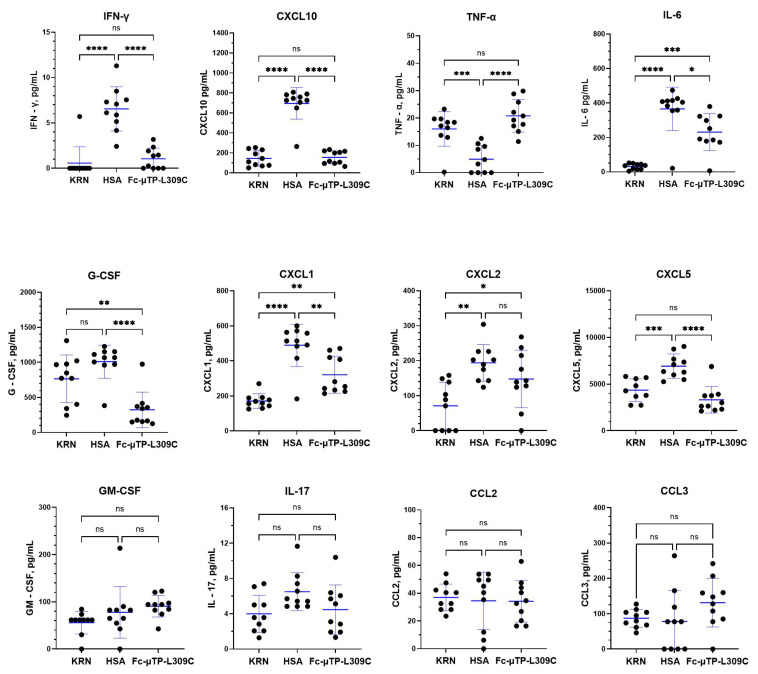
Multiplex analysis of inflammatory cytokines/chemokines in K/BxN plasma. K/BxN mice were given 6 s.c. injections on days 1, 3, 5, 7, 9, and 11 of 200 mg/kg of Fc-µTP-L309C, and HSA as protein control. Mice that developed high clinical scores of 9–12 were evaluated for cytokine/chemokines in citrated plasma, collected from peripheral blood via the saphenous vein, using multiplex analysis. Age-matched, female KRN mice served as healthy controls; *n* = 10 for each group. Multiplex cytokine analysis was performed using the Luminex platform with values determined by 5-parameter logistic regression analysis of standard curves. Cytokines with undetectable levels are reported as 0 pg/mL. Each data point represents the cytokine value for one animal. Means +/− standard deviation are shown. Groups were compared using one-way Anova with Tukey’s post hoc test; * *p* < 0.05; ** *p* < 0.01; *** *p* < 0.001; **** *p* < 0.0001; ns, not significant. IL-4 was undetected.

K/BxN mice were given six s.c. injections on days 1, 3, 5, 7, 9, and 11 of 200 mg/kg of Fc-µTP-L309C, and HSA as protein control. Mice that developed high clinical scores of 9–12 were evaluated for cytokine/chemokines in citrated plasma, collected from peripheral blood via the saphenous vein, and joint synovial fluid, using multiplex analysis. Figure 3 shows the results using plasma, where inflammatory cytokines IFN-γ, CXCL10 (IP-10), IL-6, CXCL1 (KC), CXCL2 (MIP-2) and CXCL5 (LIX) were all significantly elevated in the plasma of K/BxN mice, and treatment with Fc-µTP-L309C significantly reduced the levels of all but CXCL2 (MIP-2) (Figure 3). G-CSF was not elevated in the untreated K/BxN mice, but treatment with Fc-µTP-L309C resulted in a significant reduction in its levels. TNF-α, interestingly, was decreased in the HSA-treated mice in relation to KRN baseline levels and was elevated back to baseline with Fc-µTP-L309C treatment. No IL-4 was detectable.

**Figure 4:**
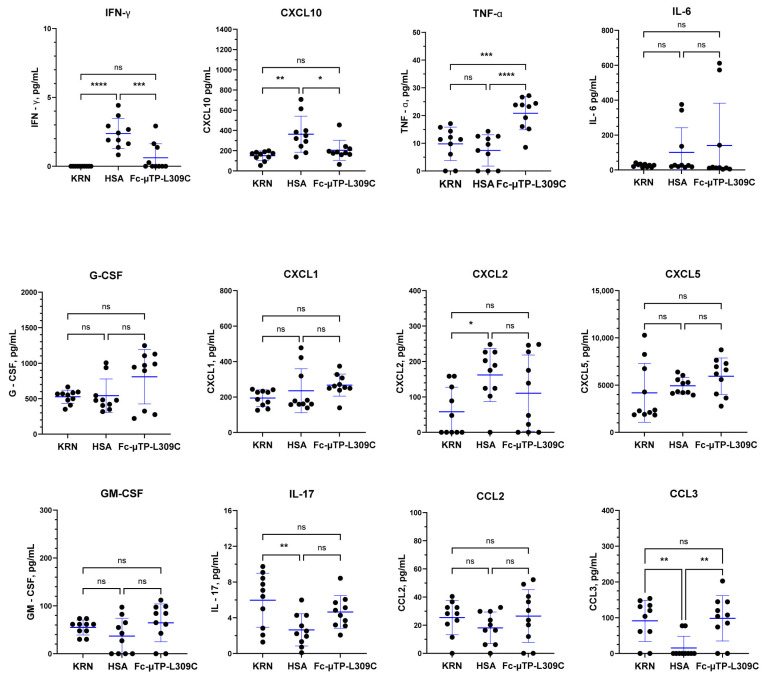
Multiplex analysis of inflammatory cytokines/chemokines in K/BxN synovial fluid. K/BxN mice were given 6 s.c. injections on days 1, 3, 5, 7, 9, and 11 of 200 mg/kg of Fc-µTP-L309C, and HSA as protein control. Mice that developed high clinical scores of 9–12 were evaluated for cytokine/chemokines in synovial fluid collected from the joints. Age-matched, female KRN mice served as healthy controls; *n* = 10 for each group. Multiplex cytokine analysis was performed using the Luminex platform with values determined by 5-parameter logistic regression analysis of standard curves. Cytokines with undetectable level are reported as 0 pg/mL. Each data point represents the cytokine value for one animal. Means +/− standard deviation are shown. Groups were compared using one-way Anova with Tukey’s post hoc test, * *p* < 0.05; ** *p* < 0.01; *** *p* < 0.001; **** *p* < 0.0001; ns, not significant. IL-4 was undetected in all samples.

Figure 4 shows the results obtained for synovial fluid. IFN-γ and CXCL10 (IP-10) were significantly decreased as seen in the plasma, but no significant differences were seen with IL-6, G-CSF, CXCL1 (KC), CXCL2 (MIP-2), or CXCL5 (LIX). CXCL2 (MIP2) was increased in HSA control relative to KRN and showed a trend downwards with Fc-µTP-L309C treatment, although this was not significant. TNF-α, as with plasma, was significantly increased with Fc-µTP-L309C treatment. IL-17 and CCL3 (MIP-1α), however, unlike in the plasma, showed a decrease from KRN baseline with HSA, but not with Fc-µTP-L309C. Levels for IL-4 were undetectable.

### 2.4. MCP-5, IL-11 and TGF-β1 Are Decreased in the Joint Following Fc-µTP-L309C Treatment

**Figure 5:**
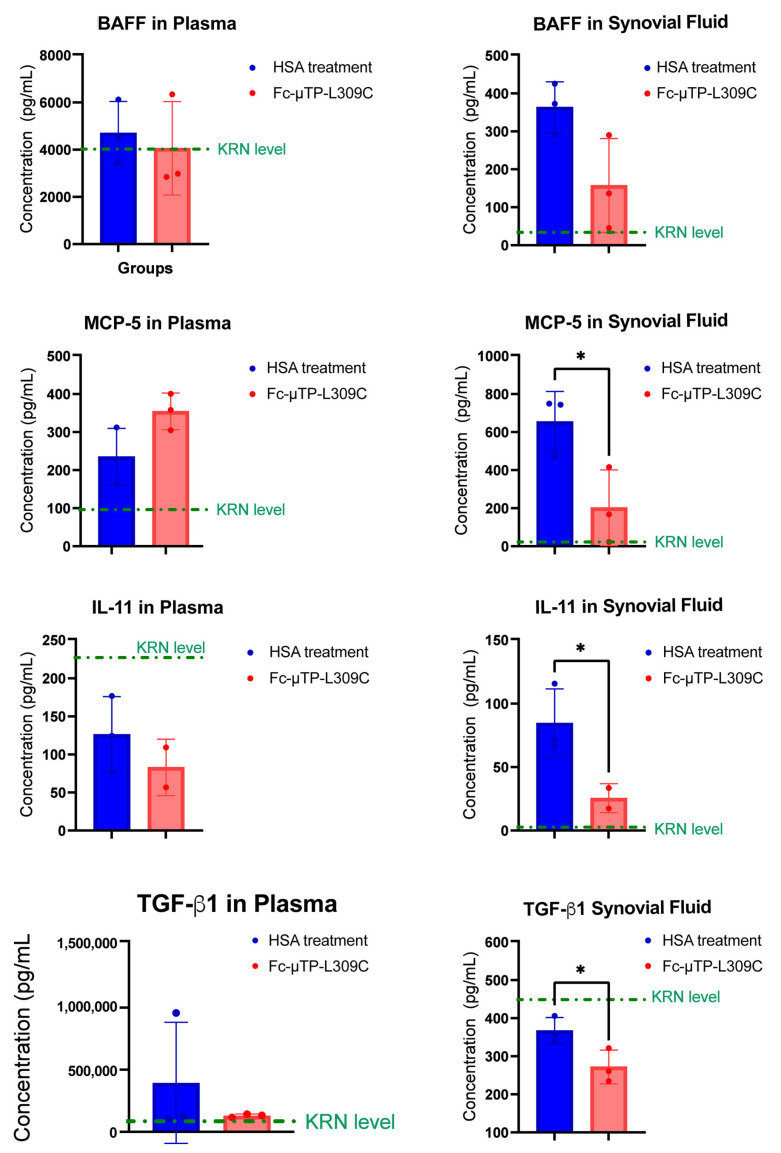
Levels of B-cell regulatory cytokines in K/BxN mice treated with Fc-µTP-L309C. K/BxN mice were given 6 s.c. injections on days 1, 3, 5, 7, 9, and 11 of 200 mg/kg of Fc-µTP-L309C, and HSA as protein control. Mice that developed high clinical scores of 9–12 were evaluated for cytokine/chemokines in citrated plasma, collected from peripheral blood via the saphenous vein, and joint synovial fluid, using multiplex analysis tested by the Luminex platform for BAFF, MCP-5, IL-11 and TGF-β1. Results represent the mean +/− SD of 3 mice using Student’s *t*-test. * *p* < 0.05.

We next performed preliminary testing of cytokines/chemokines specifically involved in the regulation of B-cells (Figure 5). K/BxN mice were given six s.c. injections on days 1, 3, 5, 7, 9, and 11 of 200 mg/kg of Fc-µTP-L309C, and HSA as protein control. Mice that developed high clinical scores of 9–12 were evaluated for cytokine/chemokines in citrated plasma, collected from peripheral blood via the saphenous vein, and joint synovial fluid, using Luminex analysis, to evaluate the cytokine/chemokines involved in the regulation of B-cells. MCP-5, IL-11 and TGF-β1 were all significantly decreased in the joint fluid after treatment with Fc-µTP-L309C. There were no significant changes in these cytokines in the peripheral blood, although IL-11 and TGF-β1 showed a trend of downregulation and MCP-5 showed a trend for increased levels. BAFF levels in peripheral blood did not change; however, there was a decrease in the joint fluid after Fc-µTP-L309C treatment compared to HSA, from mean value of 350 pg/mL to 150 pg/mL; however, this did not reach statistical significance.

## 3. Discussion

We observed that upon repeated administration of Fc-µTP-L309C in K/BxN mice, the anti-G6PI concentration decreased in the peripheral blood relative to concentrations seen in the HSA injected control (Figure 1A). We hypothesized that this could happen through the binding of Fc-µTP-L309C to FcRn or through some effect of Fc-µTP-L309C on B-cells via interaction with FcγRIIb, to reduce Ab production. In the K/BxN serum-transfer model, arthritis develops in normal mice following the transfer of anti-G6PI Abs that complex with endogenous G6PI [17]. These ICs infiltrate joints, where they initiate an inflammatory cascade within minutes following transfer [8,9,18]. This model is therefore instructive for the analysis of therapies that target the humoral response.

We first investigated the effects of Fc-µTP-L309C on FcRn. FcRn protects IgG from degradation, and it extends the serum half-life of IgG by recycling it. This is especially important in IgG-mediated autoimmune conditions where auto-Abs are propagated throughout the body by FcRn to enhance pathogenicity [19,20,21]. Enhanced degradation of auto-Abs and alleviation of autoimmune symptoms have been demonstrated after treatment of animals with molecules competing for binding of IgG to FcγRn [22], and such molecules, like the hIgG1-Fc fragment with enhanced affinity binding to FcRn or mAbs against FcRn, are currently registered on the market for treatment of immune thrombocytopenia and myasthenia gravis or in clinical development for other indications, e.g., chronic inflammatory demyelinating polyneuropathy or Sjögrens Disease [23,24,25].

We have previously shown that Fc-µTP-L309C bound to human FcRn with high avidity, and so it may block recycling of IgG through FcRn, thereby increasing autoantibody degradation [4]. Since arthritis in the K/BxN serum-transfer model is dependent on the availability of pathogenic antibodies and FcRn is the receptor primarily responsible for extending IgG life span, we examined whether Fc-µTP-L309C was able to ameliorate arthritis in FcRn KO mice using the K/BxN serum-transfer model. It should be noted that FcRn KO mice are generally resistant to serum-transfer-induced arthritis. However, the protective effect of an FcRn-deficiency could be partially overcome when larger doses (500 or 1000 μL) of K/BxN serum are transferred. In this study, we found that transferring larger doses of K/BxN serum (1000 μL) to FcRn KO mice did induce arthritis; however, Fc-µTP-L309C was able to ameliorate arthritis in these mice (Figure 1B). As a complimentary study, we also showed that when the H310L mutation is introduced to Fc-µTP-L309C, which abrogates binding to FcRn, this molecule (Fc-µTP-L309C-H310L) is still able to ameliorate arthritis after repeated administration in K/BxN mice with high clinical scores (Figure 1C). These results show that Fc-µTP-L309C is not critically dependent on FcRn for its ability to ameliorate arthritis in K/BxN mice and that, instead, it likely involves a collaboration of various other mechanisms of action.

As the reduction in autoantibody production was not related to the FcRn, we next investigated the effects of Fc-µTP-L309C on B-cell antibody production. Arthritis progression in K/BxN mice is driven by activation of T-cells expressing the KRN TCR that recognizes G6PI bound to the NOD-derived I-Ag [6,7,18,26] molecule on MHC class II APCs [26,27]. Activated T-cells subsequently interact with B-cells through TCR: Ag [7]–MHC class II molecules and CD40:CD40L engagement, thereby promoting polyclonal B-cell activation and T-helper cell-dependent production of disease-inducing IgGs [5,7,9,28,29].

FcγRIIb is the only FcγR expressed on B-cells and liver sinusoidal endothelial cells, and it mediates an inhibitory signal when bound by IgG to stop antibody production by B-cells [26,27]. We have previously shown that Fc-µTP-L309C binds to human FcγRIIb with high avidity, and so it may reduce Ab production by B-cells, thereby decreasing the number of pathogenic auto-Abs available [4]. We did indeed show that B-cells in the spleens and popliteal lymph nodes of K/BxN mice that had been repeatedly administered Fc-µTP-L309C decreased anti-G6PI production and that there was subsequently less IgG deposition on the ankle joints of K/BxN mice (Figure 2B,C). Although Fc-µTP-L309C did not modulate FcγRIIb expression in K/BxN mice (Figure 2A), we cannot rule out an effect on FcγRIIb. The Fc hexamer might be bound to B-cells, masking the epitope recognized by the staining antibody; and, therefore, no difference is observed. Thus, activation of the ITIM-motif inhibitory FcγRIIb may have occurred and is responsible for or contributes to the lack of GPI antibody production. Further cellular studies are needed to better understand the binding kinetics of Fc-µTP-L309C to mouse B-cells derived from the spleen.

We have previously shown that Fc-µTP-L309C ameliorates arthritis through multiple effects on neutrophils and T-cells [4,14,15,16]. We now show that a major effect of Fc-µTP-L309C is via effects in the humoral immune system. We show here that Fc-µTP-L309C can directly downregulate specific G6PI autoantibody secretion from B-cells, reducing the systemic level of the antibody, independent of FcRn. We also found that K/BxN mice have, as expected, significantly increased inflammatory molecules that can be downregulated by Fc-µTP-L309C treatment (Figure 3 and Figure 4). Fc-µTP-L309C also had significant effects on cytokines involved in B-cell differentiation and antibody production.

Our results when testing for 13 inflammatory cytokines/chemokines showed, for the first time using the endogenous K/BxN mouse model of arthritis, that the inflammatory cytokines IFN-γ, CXCL10 (IP-10), IL-6, CXCL1 (KC), CXCL2 (MIP-2) and CXCL5 (LIX) were all significantly elevated in the plasma of K/BxN mice, and treatment with Fc-µTP-L309C significantly reduced the levels of all but CXCL2 (MIP-2) (Figure 3). G-CSF was not elevated in the untreated K/BxN mice, but treatment with Fc-µTP-L309C resulted in a significant reduction in its levels. TNF-α, interestingly, was decreased in the HSA-treated mice, and levels were raised back to baseline with Fc-µTP-L309C treatment. This fits with a previous report that a neutralizing anti-TNF-α had no effect in preventing arthritis development in this mouse model [30]. Similar results were found in synovial fluid (Figure 4) with effects in lowering IFN-γ and CXCL10 (IP-10), but no significant differences apparent for IL-6, G-CSF, CXCL1 (KC), CXCL2 (MIP-2), or CXCL5 (LIX). CXCL2 (MIP2) was increased in HSA control and showed a trend downwards with Fc-µTP-L309C, although it was not significant. TNF-α, as with plasma, was significantly increased with Fc-µTP-L309C treatment. IL-17 and CCL3 (MIP-1α), however, unlike in the plasma, showed a decrease from KRN baseline with HSA treatment, but not with Fc-µTP-L309C.

IL-11 and TGF-β1 were found to be downregulated in both peripheral blood and synovial fluid, while MCP-5 was slightly up-regulated in peripheral blood and significantly downregulated in the synovial fluid with Fc-µTP-L309C treatment (Figure 5). These preliminary results require follow-up but provide some insight into the possible mechanism of inhibition of anti-GPI antibody production from B-cells. IL-11 is known to affect B-cells, as it can promote B-cell differentiation, stimulate IgG production, and support B-cell development and function in vitro and in vivo. IL-11 receptor expression has also been found on human B-cells, indicating a direct interaction [30]. TGF-β1 significantly affects B-cells by inhibiting their proliferation, promoting their apoptosis, and influencing their differentiation, particularly promoting IgA isotype switching while hindering IgG production [31]. MCP-5 can affect B-cells by attracting them to inflammatory sites, a process crucial for the host’s immune response [32]. BAFF can significantly affect B-cells by supporting their survival, maturation, and differentiation through interactions with its receptors (BAFF-R, TACI, and BCMA) on B-cell surfaces. It helps regulate B-cell homeostasis by preventing apoptosis in naive and memory B-cells, promoting immunoglobulin class switching, and contributing to the development of the mature peripheral B-cell population [33]. Reductions in BAFF can also lead to a diminished ability to mount an antibody response [34]. Although there was no effect of Fc-µTP-L309C on BAFF levels in peripheral blood, a trend was apparent for downregulation in the synovial fluid. These effects of Fc-µTP-L309C require further study and confirmation, but could explain the FcRn-independent lowered levels of anti-G6PI found in the study. Use of additional animal models of endogenous autoantibody production may show that the effect of Fc-µTP-L309C applies to antibody secretion in general. Future studies should further investigate Fc-µTP-L309C as a potential therapeutic option for antibody-mediated autoimmune diseases.

## 4. Materials and Methods

### 4.1. Mice

KRN TCR transgenic mice on a C57BL/6 background were obtained from the Jackson Laboratory, a kind gift from C. Benoist. NOD/Lt mice were purchased from the Jackson Laboratory. Arthritic mice were obtained by crossing KRN mice (F, 6 weeks old) with NOD/Lt (M, 6 weeks old) mice to produce K/BxN mice expressing both the TCR transgene KRN and the MHC class II molecule I-Ag7. FcRn KO (M and F, 6 weeks old) mice and BALB/c (F 6, weeks old) mice were purchased from the Jackson Laboratory. Mice were kept under a natural light–dark cycle, maintained at 22 ± 4 °C, and fed with standard diet and water ad libitum. All experiments were performed after animal use protocols (AUP 1788) were approved by the University Health Network Animal Research Committee in Toronto.

### 4.2. Biological Reagents

Human plasma-derived 20% subcutaneous immunoglobulin (SCIG, Hizentra, CSL Behring, Bern, Switzerland), Fc-µTP-L309C, and Fc-µTP-L309C-H310L were produced by CSL Behring, Bern, Switzerland. Human serum albumin (HSA) was from the Canadian Blood Services. Recombinant mouse G6PI was provided as a kind gift from R. Holmdahl (Karolinska Institute, Stockholm, Sweden).

### 4.3. Anti-G6PI ELISA

Five µg/mL of recombinant mouse G6PI in PBS was coated on ELISA microtiter plates overnight at 4 °C. Mouse peripheral blood plasma was diluted using 10-fold serial dilutions (1:3 to 1:3,000,000) as described previously [15]. Anti-G6PI was detected with alkaline phosphatase-conjugated goat anti-mouse IgG (Jackson Immunoresearch Laboratories, Baltimore, PA, USA). The concentration of the anti-G6PI in the peripheral blood plasma was determined using rabbit anti-mouse G6PI (clone A11695; Antibodies Online, Limerick, PA, USA).

### 4.4. K/BxN Serum-Transfer Arthritis

Severely arthritic adult K/BxN mice were bled, and the sera was pooled. FcRn KO mice were injected i.p. with 200 µL of pooled sera on days 0 and 2 as indicated in the figure legends. The volume of sera was chosen based on in vivo titration of pooled sera. Mice were given treatment on day 2 with either a s.c. injection of 200 mg/kg of Fc-µTP-L309C or a s.c. injection of 2000 mg/kg of SCIg as indicated in the figure legends. HSA was used as a protein control.

### 4.5. Arthritis Treatment in the K/BxN Mouse Model of Endogenous, Chronic Arthritis

K/BxN mice with high clinical scores of 9 or greater were treated by either s.c. injections of 200 mg/kg Fc-µTP-L309C, s.c. injections of 200 mg/kg Fc-µTP-L309C-H310L, or s.c. injections of 2000 mg/kg of SCIg as indicated in the figure legends on days 1, 3, 5, 7, 9, and 11. The H310L mutation reduces FcRn binding. However, it does not impair the formation of Fc hexamers [35]. HSA was used as a protein control.

### 4.6. Arthritis Scoring

The clinical scores of the mice were monitored daily over the course of each experiment. The development of arthritis was assessed daily as described previously [15,16,36,37], and the severity of arthritis was scored for each paw on a 3-point scale, in which 0 = normal appearance, 1 = localized edema/erythema over one surface of the paw, 2 = edema/erythema involving more than one surface of the paw, 3 = marked edema/erythema involving the whole paw. The scores of all four paws were added for a composite score, with a maximum total score of 12 per mouse.

### 4.7. Binding of Fc-µTP-L309C to FcγRIIb on B-Cells

Spleens were removed from K/BxN mice and placed in RPMI 1640 + 5% FCS on ice. The spleens were pushed through a fine mesh strainer (70 µm cutoff) to form a single-cell suspension, and RBCs were lysed. The cells were washed and resuspended in PBS + 2% FCS for cell counts. B-cells were enriched using the EasySep mouse B-cell enrichment kit (Stemcell Technologies, Vancouver, BC, Canada). Cells were incubated with 100 µg/mL of Fc-µTP-L309C for 30 min at 37 °C and stained for B220-APC (clone RA3-6B2, Biolegend, San Diego, CA, USA) and FcγRIIb-PE (clone AT130-2, Thermofisher Scientific, Waltham, MA, USA). FcγRIIb staining was analyzed by flow cytometry on a BD LSRFortessa (BD Biosciences, San Jose, CA, USA), and the data were analyzed by using FlowJo software, Version 9 (Ashland, OR, USA).

### 4.8. Anti-G6PI ELISpot

The spleens and popliteal lymph nodes from K/BxN mice that received 6 s.c. injections of 200 mg/kg of Fc-µTP-L309C were isolated and placed in RPMI 1640 + 5% FCS on ice. Mice treated with HSA were used as a control. The spleens and popliteal lymph nodes were pushed through a fine mesh strainer (70 µm cutoff) to form a single-cell suspension. RBCs were lysed from the spleens. The cells were washed and resuspended in PBS + 2% FCS for cell counts. Then, 96-well filter plates (MilliporeSigma, Burlington, MA, USA) were coated with 5 µg/mL of recombinant mouse G6PI overnight at 4 °C. Spleen cells and popliteal lymph node cells were seeded onto the plate at 10^6^ cells per well at 37 °C overnight. The cells were washed from the plate, and the wells were stained with goat anti-mouse IgG (Jackson Immunoresearch Laboratories, West Grove, PA, USA) conjugated to HRP for 2 h at 37 °C. AEC (SK-4200, Vector laboratories, Burlingame, CA, USA) was added into each well and color development could occur for 9 min until the reaction was stopped with water. The plates were left to dry at room temperature (RT) overnight and the spots on the membrane were counted and analyzed using ImmunoSpot 7.0.23.3 (San Diego, CA, USA).

### 4.9. Immunofluorescence

Dissected ankle joints from K/BxN mice that received 6 s.c. injections of 200 mg/kg of Fc-µTP-L309C were embedded in OCT, frozen in liquid nitrogen and mounted on a cryomicrotome support at −25 °C. Mice treated with HSA were used as positive controls and BALB/c mice were used as negative controls. Sagittal sections (6–8 µm thick) were cut and transferred to an adhesive coated slide. Slides were stored at −80 °C until use and then acetone-fixed for 1 min and air dried for 30 min. The deposition of IgG was detected by Alexafluor488-conjugated goat anti-mouse IgG (Jackson Immunoresearch Laboratories). Nuclei were counterstained with DAPI (Thermofisher Scientific). Representative images were taken using the LeicaSP8/STED Confocal microscope (Leica Camera AG, Wetzlar, Germany) and the images were analyzed using ImageJ (Bethseda, MD, USA).

### 4.10. Cytokines

Cytokine kits used in the study: Mouse Cytokine MAGNETIC Kit (GM-CSF, G-CSF, IFN-γ, IL-4, IL-6, IP-10, IL-17, KC (CXCL1), MCP-1 (CCL2), MIP-1α (CCL3), MIP-2 (CXCL2), LIX (CXCL5), TNF-α), Millipore-Sigma multiplex Catalog #MCYTOMAG-70K-13. Millipore-Sigma in the U.S. and Canada. Mouse Cytokine/MC2 (IL-11, MCP-5). Multiplex Catalog # MECY2MAG-73K-02. Millipore-Sigma in the U.S. and Canada. MILLIPLEX TGF-beta 1,2,3 MAGNETIC Bead Kit. Millipore-Sigma in the U.S. and Canada. Mouse XL Cytokine Base Kit, BAFF/BLyS/TNFSF13B XL (Catalog Number LMXL000, R&D Systems^®^, Bio-Techne, Toronto, ON, Canada). Cytokines were measured using a Luminex platform in synovial fluid taken from the joints of K/BxN mice or from peripheral blood citrated plasma taken via the saphenous vein after treatment with 3–6 s.c. injections of 200 mg/kg of rFc-µTP-L309C. KRN mice and mice treated with HSA were used as a control.

### 4.11. Joint Washes

Joint washes were performed as previously described [38]. Malleoli and surrounding soft tissue (excluding fat) were removed from both rear limbs and placed in RPMI 1640 + 5% FCS on ice for 60 min. The medium was then removed, centrifuged, and the supernatant (joint wash) stored at −20 °C until subsequent analysis.

### 4.12. Statistical Analyses

Statistical tests were performed using GraphPad Prism 8 for Windows software. Analyses of differences between sample groups were performed using the tests indicated in the figure legends. Data shown are mean ± standard deviation (SD), unless otherwise stated. *p* < 0.05 was considered statistically significant.

## Data Availability

The raw data supporting the conclusions of this article will be made available by the authors on request.
